# Supplementary material for: HIVprotI: an integrated web based platform for prediction and design of HIV proteins inhibitors
Source: J Cheminform. 2018 Mar 9;10:12. doi: 10.1186/s13321-018-0266-y (PMC5845081; doi:10.1186/s13321-018-0266-y)
Supplement: Supplementary file 2 — Additional file 2. Source code of HIVProtI web server. [file 13321_2018_266_MOESM2_ESM.zip › HIVprotI_Source-code/hivprotI/weka-3-6-6/doc/weka/gui/visualize/JPEGWriter.html]

JPEGWriter


---


|  |  |  |
| --- | --- | --- |
| **Overview** \| **Package** \| **Class** \| **Tree** \| **Deprecated** \| **Index** \| **Help** \| **Weka's home** | |  |
| **PREV CLASS**   **NEXT CLASS** | **FRAMES**    **NO FRAMES**     **All Classes** |  |
| SUMMARY: NESTED \| FIELD \| CONSTR \| METHOD | DETAIL: FIELD \| CONSTR \| METHOD |  |


---


## weka.gui.visualize Class JPEGWriter

```
java.lang.Object
  weka.gui.visualize.JComponentWriter
      weka.gui.visualize.JPEGWriter
```

---

``` public class JPEGWriter extends JComponentWriter ```

This class takes any JComponent and outputs it to a JPEG-file.
Scaling is by default disabled, since we always take a screenshot.

**Version:**
:   $Revision: 7059 $

**Author:**
:   FracPete (fracpete at waikato dot ac dot nz)

---

**Constructor Summary**

`JPEGWriter()`
  
          initializes the object.

`JPEGWriter(javax.swing.JComponent c)`
  
          initializes the object with the given Component.

`JPEGWriter(javax.swing.JComponent c,
java.io.File f)`
  
          initializes the object with the given Component and filename.


| **Method Summary** | |
| --- | --- |
| `void` | `generateOutput()`             generates the actual output. |
| `java.awt.Color` | `getBackground()`             returns the current background color. |
| `java.lang.String` | `getDescription()`             returns the name of the writer, to display in the FileChooser. |
| `java.lang.String` | `getExtension()`             returns the extension (incl. |
| `float` | `getQuality()`             returns the quality the JPEG will be stored in. |
| `void` | `initialize()`             further initialization. |
| `static void` | `main(java.lang.String[] args)`             for testing only. |
| `void` | `setBackground(java.awt.Color c)`             sets the background color to use in creating the JPEG. |
| `void` | `setQuality(float q)`             sets the quality the JPEG is saved in. |

**Methods inherited from class weka.gui.visualize.JComponentWriter**

`getComponent, getCustomHeight, getCustomWidth, getFile, getScalingEnabled, getUseCustomDimensions, getXScale, getYScale, setComponent, setCustomHeight, setCustomWidth, setFile, setScale, setScalingEnabled, setUseCustomDimensions, toOutput, toOutput, toOutput`

**Methods inherited from class java.lang.Object**

`equals, getClass, hashCode, notify, notifyAll, toString, wait, wait, wait`

**Constructor Detail**

### JPEGWriter

```
public JPEGWriter()
```

:   initializes the object.

---


### JPEGWriter

```
public JPEGWriter(javax.swing.JComponent c)
```

:   initializes the object with the given Component.

    **Parameters:**: `c` - the component to print in the output format

---


### JPEGWriter

```
public JPEGWriter(javax.swing.JComponent c,
                  java.io.File f)
```

:   initializes the object with the given Component and filename.

    **Parameters:**: `c` - the component to print in the output format: `f` - the file to store the output in


**Method Detail**

### initialize

```
public void initialize()
```

:   further initialization.

---


### getDescription

```
public java.lang.String getDescription()
```

:   returns the name of the writer, to display in the FileChooser.
    must be overridden in the derived class.

    :   **Specified by:**: `getDescription` in class `JComponentWriter`
    :   **Returns:**: the name of the writer

---


### getExtension

```
public java.lang.String getExtension()
```

:   returns the extension (incl. ".") of the output format, to use in the
    FileChooser.
    must be overridden in the derived class.

    :   **Specified by:**: `getExtension` in class `JComponentWriter`
    :   **Returns:**: the file extension

---


### getBackground

```
public java.awt.Color getBackground()
```

:   returns the current background color.

    :   **Returns:**: the current background color

---


### setBackground

```
public void setBackground(java.awt.Color c)
```

:   sets the background color to use in creating the JPEG.

    :   **Parameters:**: `c` - the color to use for background

---


### getQuality

```
public float getQuality()
```

:   returns the quality the JPEG will be stored in.

    :   **Returns:**: the quality

---


### setQuality

```
public void setQuality(float q)
```

:   sets the quality the JPEG is saved in.

    :   **Parameters:**: `q` - the quality to use

---


### generateOutput

```
public void generateOutput()
                    throws java.lang.Exception
```

:   generates the actual output.

    :   **Throws:**: `java.lang.Exception` - if something goes wrong

---


### main

```
public static void main(java.lang.String[] args)
                 throws java.lang.Exception
```

:   for testing only.

    :   **Parameters:**: `args` - the commandline arguments **Throws:**: `java.lang.Exception` - if something goes wrong


---


|  |  |  |
| --- | --- | --- |
| **Overview** \| **Package** \| **Class** \| **Tree** \| **Deprecated** \| **Index** \| **Help** \| **Weka's home** | |  |
| **PREV CLASS**   **NEXT CLASS** | **FRAMES**    **NO FRAMES**     **All Classes** |  |
| SUMMARY: NESTED \| FIELD \| CONSTR \| METHOD | DETAIL: FIELD \| CONSTR \| METHOD |  |


---
